# Effect of heat acclimation on metabolic adaptations induced by endurance training in soleus rat muscle

**DOI:** 10.14814/phy2.14686

**Published:** 2021-08-17

**Authors:** Pierre‐Emmanuel Tardo‐Dino, Cindy Taverny, Julien Siracusa, Stéphanie Bourdon, Stéphane Baugé, Nathalie Koulmann, Alexandra Malgoyre

**Affiliations:** ^1^ Unité de Physiologie de l’Exercice et des Activités en Conditions Extrêmes Département Environnements Opérationnels Institut de Recherche Biomédicale des Armées Brétigny sur Orge France; ^2^ Ecole du Val‐de‐Grâce Paris France; ^3^ EDISS 205 Université Claude Bernard Lyon 1 Villeurbanne France; ^4^ LBEPS Université Evry IRBA Université Paris‐Saclay Paris 91025 France

**Keywords:** endurance training, heat acclimation, mitochondrial respiration, skeletal muscle metabolism cross‐tolerance

## Abstract

Aerobic training leads to well‐known systemic metabolic and muscular alterations. Heat acclimation may also increase mitochondrial muscle mass. We studied the effects of heat acclimation combined with endurance training on metabolic adaptations of skeletal muscle. Thirty‐two rats were divided into four groups: control (C), trained (T), heat‐acclimated (H), and trained with heat acclimation (H+T) for 6 weeks. Soleus muscle metabolism was studied, notably by the *in situ* measurement of mitochondrial respiration with pyruvate (Pyr) or palmitoyl‐coenzyme A (PCoA), under phosphorylating conditions (${\dot{V}}_{\text{max}}$) or not (${\dot{V}}_{0}$). Aerobic performance increased, and retroperitoneal fat mass decreased with training, independently of heat exposure (*p* < 0.001 and *p* < 0.001, respectively). Citrate synthase and hydroxyl‐acyl‐dehydrogenase activity increased with endurance training (*p* < 0.001 and *p* < 0.01, respectively), without any effect of heat acclimation. Training induced an increase of the ${\dot{V}}_{0}$ and ${\dot{V}}_{\text{max}}$ for PCoA (*p* < .001 and *p* < .01, respectively), without interference with heat acclimation. The training‐induced increase of ${\dot{V}}_{0}$ (*p* < 0.01) for pyruvate oxidation was limited when combined with heat acclimation (−23%, *p* < 0.01). Training and heat acclimation independently increased the ${\dot{V}}_{\text{max}}$ for pyruvate (+60% *p* < 0.001 and +50% *p* = 0.01, respectively), without an additive effect of the combination. Heat acclimation doubled the training effect on muscle glycogen storage (*p* < 0.001). Heat acclimation did not improve mitochondrial adaptations induced by endurance training in the soleus muscle, possibly limiting the alteration of carbohydrate oxidation while not facilitating fatty‐acid utilization. Furthermore, the increase in glycogen storage observed after HA combined with endurance training, without the improvement of pyruvate oxidation, appears to be a hypoxic metabolic phenotype.

## Graphical abstract

We studied the consequences of heat acclimation combined with endurance training on metabolic adaptations of skeletal muscle. Heat acclimation (HA) did not improve training‐induced adaptations of mitochondrial maximal oxidation capacities for carbohydrates (Vmax PYR) or fatty acids (Vmax PCOA) in the soleus. Despite this lack of facilitation for CHO oxidation, we observed a synergistic effect of heat and endurance training on glycogen storage which may be beneficial for the glycolytic pathway. This adresses the issue of this combined conditioning as an alternative in preparattion for sports requiring glycogen storage or glycoltyic flux.

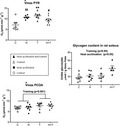

## 1 INTRODUCTION

Heat acclimation and endurance training result in increased endurance in the heat by improving the thermoregulatory response (Periard et al., 2015). Other physiological adaptations required for aerobic performance, such as cardiovascular function, are also improved by heat acclimation (Periard et al., 2016). At the cellular level, heat shock proteins (HSP) have been implicated in certain benefits related to endurance training (Henstridge et al., 2016). In addition, each strain factor also has its own signaling pathway. As described in heart muscle, heat acclimation and endurance training induce different alterations of myosin isoforms (Diffee et al., 2001; Horowitz et al., 1986) and intracellular calcium regulation (Cohen et al., 2007; Diffee et al., 2001; Mirit et al., 1999; Wisløff et al., 2001), with potentially different consequences on muscle contraction and relaxation. Most studies concerning metabolic alterations have focused on the acute effects of heat exposure during exercise (Febbraio et al., 1994; Gagnon et al., 2020; Hargreaves et al., 1996; Maunder et al., 2020; Young et al., 1985). The time courses for such studies were very short (8 days) and not sufficiently long to observe the combined effect with endurance training on muscle metabolism (Febbraio et al., 1994; King et al., 1985). Thus, metabolic adaptations induced by heat acclimation and endurance training may interfere with each other, resulting in a new phenotype that is yet to be described (Kodesh et al., 2011).

The skeletal muscle adaptations induced by aerobic training are well known. Muscle mitochondrial content has been shown to increase after endurance training (Holloszy, 1967; Holloszy & Coyle, 1984; Zoladz & Grassi, 2015; Zoll et al., 2003), involving both the size and number of mitochondria (Hoppeler et al., 1973; Morgan et al., 1971). Endurance training may also induce alterations in mitochondrial dynamics. Near to biogenesis, mitochondrial turn‐over through autophagy mechanisms could be enhanced. Furthermore, fission and fusion pathways seem to be modified after endurance training, allowing to improve mitochondrial quality (Tanaka et al., 2020). An increase in the level of certain mitochondrial enzymes enhances both carbohydrate and fatty‐acid oxidation. Indeed, endurance training increases intramuscular glycogen and triglyceride stores (Greiwe et al., 1999; Jeukendrup et al., 1998). Moreover, fatty‐acid oxidation enzyme levels specifically increase (Brooks & Mercier, 1994; Saltin et al., 1993), contributing to the relative enhancement of fatty‐acid oxidation (Brooks & Mercier, 1994), allowing the sparing of carbohydrate stores, known to be a limiting factor of performance (Lundby & Jacobs, 2016; Molé et al., 1971). Thus, endurance training induces quantitative mitochondrial adaptations, which improve their oxidative capacity, as well as qualitative adaptations, which alter the carbohydrate‐lipid balance (Brooks & Mercier, 1994).

Several studies have shown that chronic heat exposure may improve endurance at room temperature (Lorenzo & Minson, 2010; Scoon et al., 2007), suggesting that adaptations other than thermoregulation may occur. Heat has been shown to stimulate the Akt pathway, which is responsible for protein synthesis (Liu & Brooks, 2012) and may prevent muscle atrophy induced by aging, immobilization, or injury (Hafen et al., 2018; Ihsan et al., 2019; Tamura & Hatta, 2017; Tamura et al., 2015). Very recently, heat appeared to preserve the force of the soleus muscle by enhancing myogenic factors in a model of ischemia‐induced muscle damage (Kim et al., 2019). Overexpression of HSP 70 has been shown to reduce age‐related oxidative stress in muscle (Broome et al., 2006). In addition, heat acclimation may also induce endurance‐like mitochondrial adaptations. Although acute heat exposure induces an increase in carbohydrate utilization, interpreted as a higher reliance on CHO oxidation (Febbraio, 2000; Febbraio et al., 1994, 1996; Fink et al., 1975; Gagnon et al., 2020; Hargreaves et al., 1996; Maunder et al., 2020; O'Hearn et al., 2016; Young et al., 1985), a few studies have shown that repeated heat exposure may restore lipid oxidation and limit the reliance on CHO oxidation, as observed with training (King et al., 1985; Kirwan et al., 1987). Indeed, several studies have suggested that chronic heat exposure may improve oxidative metabolism. Tamura et al. (2014) showed an increase in skeletal muscle citrate synthase (CS) activity and mitochondrial respiratory chain complex content in mice exposed to 40°C for 30 min daily, 5 days/week, for 3 weeks. In addition, the combined effect of endurance training and heat was greater than that of each alone. Mechanisms remain largely unknown but the alteration of mitochondrial mass and remodeling process to preserve mitochondrial quality are also proposed. Daily heat exposure also prevented skeletal muscle insulin resistance induced by a high‐fat diet in rats, restoring glucose uptake and CS and cytochrome oxidase activities (Gupte et al., 2009). However, these results have not been confirmed by studies of mitochondrial respiration after heat acclimation. Moreover, quite surprisingly, although heat exposure has been reported to increase carbohydrate utilization, the capacity of skeletal muscle mitochondria to oxidize fatty acids has been poorly studied and never after prolonged exposure, even though Kodesh & Horowitz (2010) reported the upregulation of fatty‐acid metabolism genes in soleus muscle.

Thus, we hypothesized metabolic adaptations based on mitochondrial alterations induced by endurance training may be improved by combining it with heat acclimation. Here, we studied the individual and combined effects of endurance training and heat acclimation on aerobic performance, muscle metabolism, and mitochondrial oxidation of various substrates. We measured the effect of heat acclimation and endurance training on carbohydrate and fatty‐acid utilization in permeabilized fibers of the soleus muscle, which have a slow‐twitch phenotype, well recruited during treadmill running and sensitive to heat stress (Brownstein et al., 2017; Ganesan et al., 2016, 2018; Montilla et al., 2014; Oishi et al., 2002).

## 2 MATERIALS AND METHODS

### 2.1 Animals and experimental design

This study was performed in accordance with both the Helsinki Declaration concerning the treatment of laboratory animals and the European Convention for the Protection of Vertebrate Animals used for Experimental and other Scientific Purposes (Council of Europe no.129, Strasbourg, France, 1985 and Directive 2010/63/UE). It was approved by the animal ethics committee of our Biomedical Research Institute and authorized by the Veterinarian Inspection of Health Service for Armed Forces.

The study consisted of a 6‐week conditioning period, including training and heat acclimation. The resting metabolism (RM) and maximal aerobic speed (MAS) were measured at the end of the conditioning. Animals were then sacrificed. Both soleus (SOL) muscle, blood, and retroperitoneal adipose tissue (RPAT) were harvested. In situ mitochondrial respiration was measured in the soleus muscle. Enzymatic activity and biochemical measurements were performed on the soleus muscle and plasma.

We previously used eight animals implanted with a temperature‐monitoring device to test the protocol of heat acclimation, during a pilot study. The animals were implanted in the abdominal cavity a pill to record and visualize online the evolution of core temperature during the protocol. They were placed in a custom‐built box, which could elevate inner air temperature to 39°C. We observed an elevation of the core temperature of all animals over 38°C for 55 min and up to 39°C for 45 min.

Here, we used 32 other male Wistar rats (mean body weight: 276 ± 50 g; 8 weeks old at the beginning of the conditioning) obtained from Charles River Laboratories. Animals were housed two per cage in an artificial 12–12 light‐dark cycle and in laboratory conditions (22°C, 40% relative humidity). After a quarantine of 10 days, all rats followed a 1‐week procedure to become habituated to the treadmill. They were then randomly assigned to a condition, without changing the previous cage repartition, resulting in four groups of eight animals: control (C), heat acclimated (H), trained (T), and heat acclimated+trained (H+T). The animals were weighed weekly and at the end of the conditioning. After receiving a lethal dose of pentobarbital (150 mg kg^−1^), the two soleus muscles were excised immediately after the respiratory arrest of the animals, but before cardiac arrest, and weighed. The left soleus muscle was immediately plunged in solution S (composition see below) to preserve its vitality for mitochondrial respiration experiments and the right soleus muscle was frozen in liquid nitrogen for later measurements (enzymology, glycogen, and western‐blot measurements). Then, total exsanguination was performed by the catheterization of the abdominal aorta. Finally, retroperitoneal adipose tissue was totally removed and weighed.

### 2.2 Experimental conditions

**PHOTO 1:**
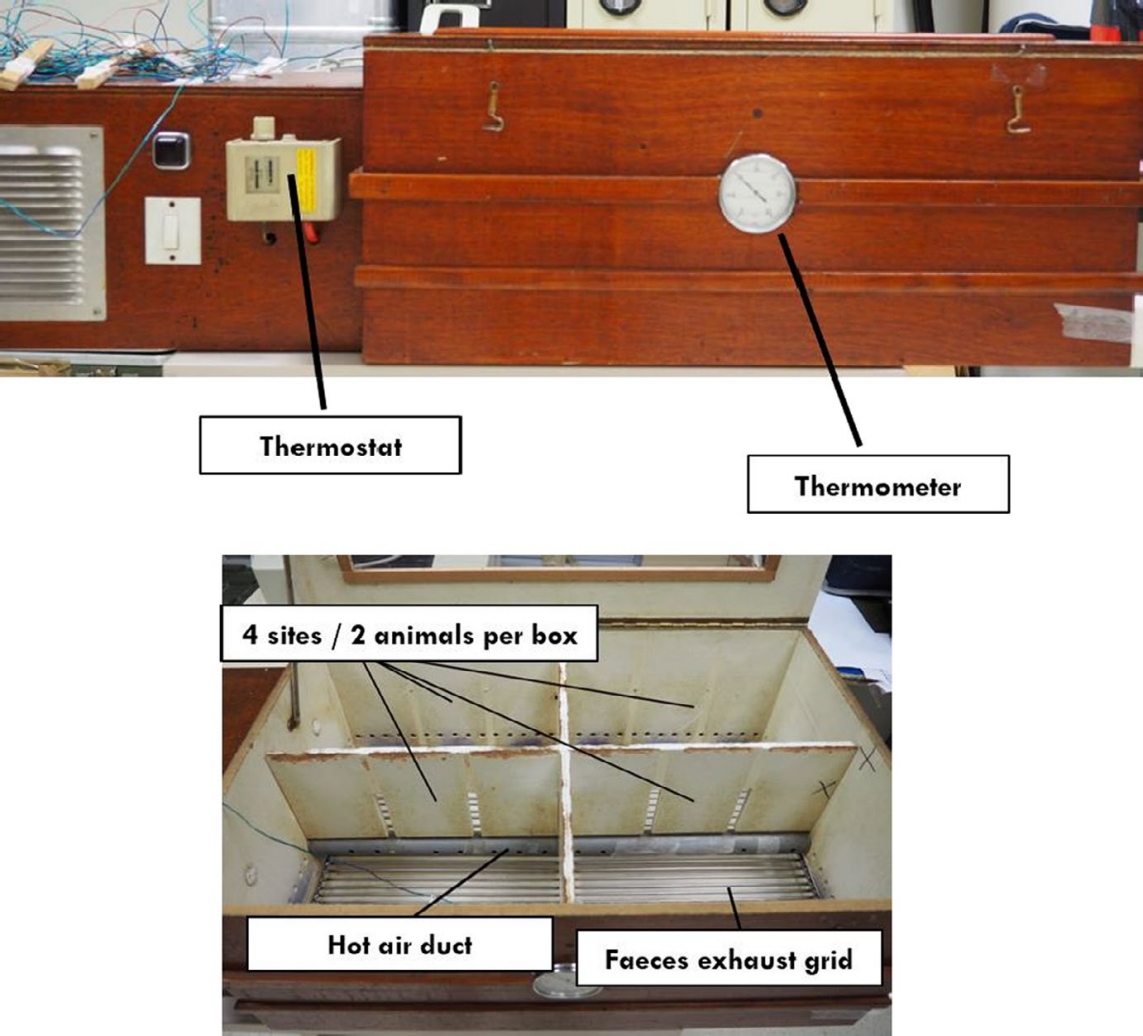
Temperature‐controlled device to perform acclimation conditioning

**FIGURE 1:**
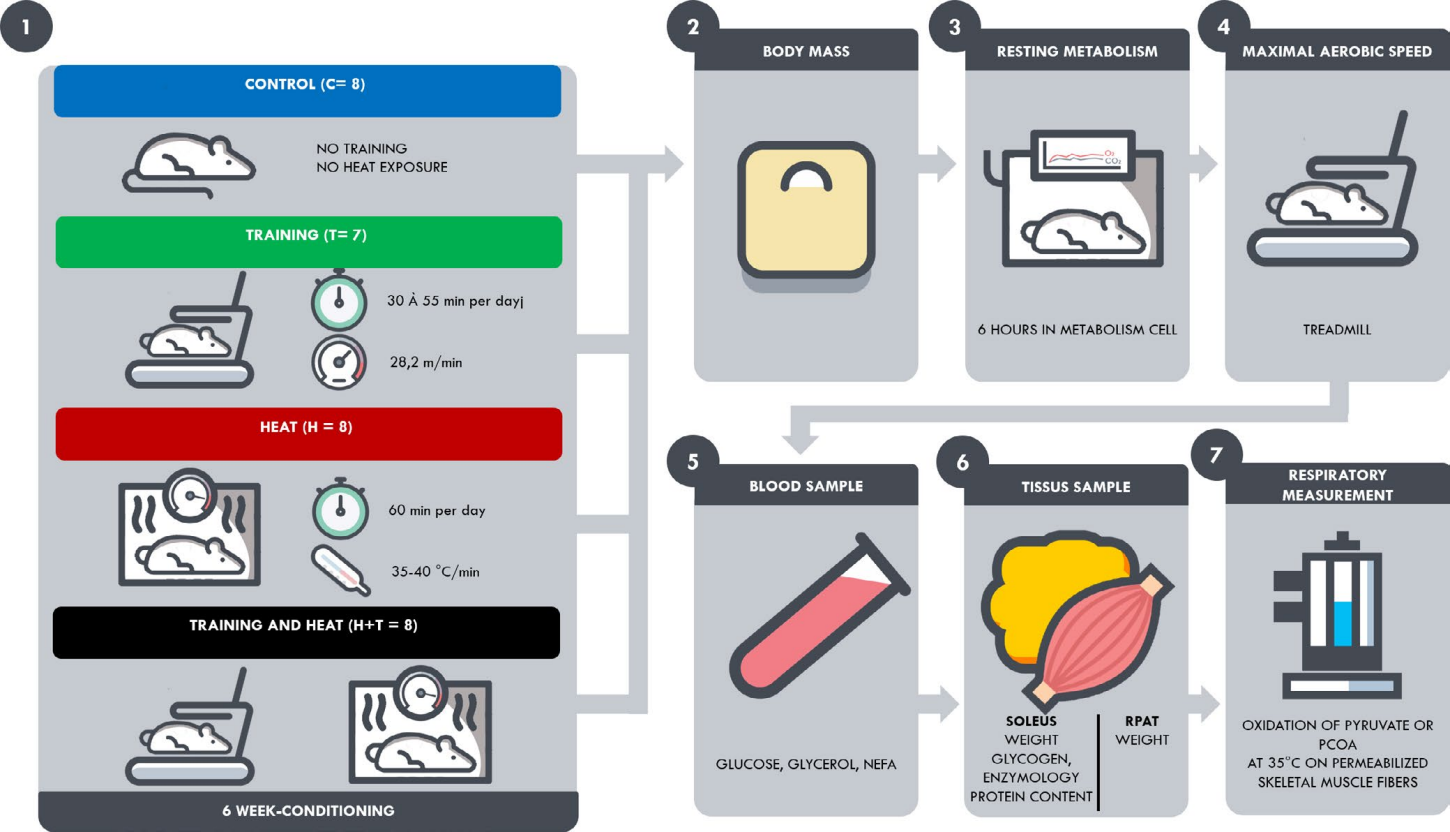
Design of experiment conditioning and steps

Heat acclimation was studied in 16 animals (H and H+T). They were exposed to 1 hr of heat, 5 days per week, for 6 weeks. The procedure consisted of placing them in the temperature‐controlled box in which the mean ambient temperature was maintained at 37 ± 3°C (Photo 1). Exposure was always performed at 2 p.m. (Figure 1).

**FIGURE 2:**
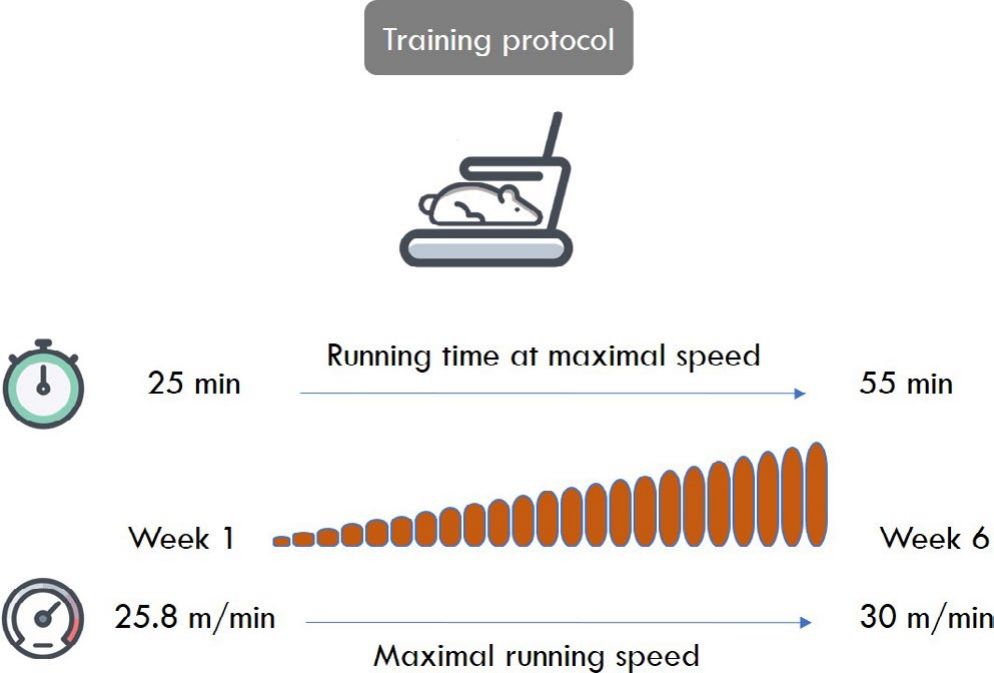
Training protocol

Sixteen animals (T and H+T) followed a daily 1‐hr endurance‐training session, 5 days per week, for 6 weeks, on a treadmill Techmachine^®^ at 9 a.m. In each session after 5 min of warm‐up around 16 m min^−1^ treadmill speed, running speed was increased in 15 min to the targeted stage. Intensity and duration progressively increased each week from a stage of 25 min at 25.8 m min^−1^ to a stage of 55 min at 30 m min^−1^ for the last week, with no slope (Figure 2). Thus, the total exercise duration was 45 min the first week up to 80 min at the last week.

### 2.3 Measurement of aerobic performance

Maximal aerobic running speed (MAS) was assessed using an incremental test. Tests were performed on the last day of conditioning and 48 hr before the animals were euthanized to prevent any interference of acute exercise with the respiratory measurements. The protocol consisted of increasing the speed by steps of 3.5 m min^−1^ each 2 min for up to 30 m min^−1^, and then steps of 1.5 m min^−1^ each 1.5 min until the rats were no longer able to run. Exhaustion was determined when the animals remained near the electric grid, despite receiving three shocks. MAS corresponded to the running speed at the last accomplished stage.

### 2.4 Resting metabolic rate

Resting metabolism was assayed using an open‐circuit indirect calorimetry device (Metabolism Software, Panlab Harvard Apparatus) the day after the performance test (the day before euthanasia). Animals were placed in a metabolic chamber that was regularly flushed with fresh air at a flow rate between 0.9 and 1.2 L/min (LE400 Air Supply and Switching, Panlab Harvard Apparatus) and connected to gas analyzers for oxygen and carbon dioxide measurements (LE405 O2/CO2 analyzer, Panlab Harvard Apparatus). Measurements were performed on fasted animals, deprived of food from 8 a.m., that is, long after the previous evening meal at a temperature of 22°C. They had free access to water but not food during the measurements. Habituation was performed during the previous days to limit transfer‐induced stress on the animals. Measurements were performed diurnally (the resting period for rodents), between 9 a.m. and 4 p.m. to limit spontaneous activity, based on a previous study (Malgoyre et al., 2017).

Data for the first hour and last 30 min were systematically excluded to avoid artifacts induced by manipulation or human presence. Moreover, a steady‐test period was defined as the longest period for which O_2_ consumption varied by <10%. O_2_ consumption was normalized to body weight raised to a power of 0.75 (Keesey & Powley, 2008).

### 2.5 Biochemical measurements

#### 2.5.1 Blood chemistry

Concentrations were determined using a colorimetric method, measuring the absorbance with an ADVIA 1800 Clinical Chemistry System (Siemens). The glucose concentration was measured after glucose oxidation by glucose oxidase, using a GLUO kit (Siemens Healthcare Diagnostics), the NEFA concentration using an RX MONZA FA 115 kit (Randox Laboratories), and the glycerol concentration using an RX MONZA GY 105 kit (Randox Laboratories).

***Enzyme activities*** were determined for citrate synthase (CS), 3‐hydroxyl‐acyl‐coenzyme A‐dehydrogenase (3‐HAD), hexokinase (HK), and lactate‐dehydrogenase (LDH). For the measurement of each activity, approximately 10 mg of frozen tissue was weighed and vortexed in a Precellys^®^ (Precellys 24 Dual, Bertin, Montigny le Bretonneux, France) at 5,000 rpm for 2 × 8 s in different extraction buffers and kept on ice at each step.

For CS, the extraction was performed in a buffer (50 mg ml^−1^) containing 5 mM HEPES (pH 8.7), 1 mM EGTA, 1 mM DTT, 5 mM MgCl_2_, and 0.1% Triton X‐100 and the samples incubated for 60 min at 0°C to ensure complete enzyme extraction. CS activity was assessed at 40°C (pH = 7.5) by the spectrophotometric measurement of the apparition of mercaptide anion (λ = 412 nm) after the addition of oxaloacetate (50 mM) (O4126 Sigma), as previously described by Srere (1969).

For 3‐HAD, the extraction was performed in 300 mM phosphate buffer (50 mg ml^−1^) containing KH_2_PO_4_, K_2_HPO_4_ (pH 7.7), and 0.05% BSA. Enzyme activity was determined at 40°C by the spectrophotometric measurement of the disappearance of NADH (λ = 340 nm) after the addition of aceto‐acetyl CoA (Sigma) in phosphate buffer (pH 7.5), containing 40 mM imidazole and 200 mM EDTA, as described by Seidemann et al. (1972).

For HK, extraction was performed in 300 mM phosphate buffer (pH 7.7) containing 5% BSA. Enzyme activity was assessed at 25°C by the spectrophotometric measurement of the appearance of NADH (λ = 340 nm) in a mix containing 100 mM Tris‐HCl (pH 8), 8 mM MgCl2, 0.4 mM NADP, 0.01% DTT, 2 mM glucose, and 3.5 UI/ml of G6PDH after the addition of ATP, as described by Lowry and Passonneau (Drake et al., 2016).

For LDH, the extraction procedure was the same as that for CS. Enzyme activity was assessed by the transformation of pyruvate into lactate, with concomitant oxidation of NADH. The disappearance of NADH (λ = 412 nm) was measured by spectrophotometry at 25°C in phosphate buffer (pH 7.5) containing 0.63 M pyruvate sodium (P2256, Sigma).

Enzyme activities are all measured as DO variation per minute and the calculation was performed taking into account the dilution of the sample and the results expressed as IU per gram of wet weight.

#### 2.5.2 Glycogen content of the soleus muscle

Frozen soleus muscle samples (5 mg) were homogenized (1/50) in 2 N NaOH for 2 hr at 37°C and 1 hr at 4°C and 0.2 volumes 7.5 M HCl added. Samples (50 µl) were subjected to glycogen hydrolysis by amyloglucosidase (10 mg/ml) (A7420, Sigma‐Aldrich) in acetate buffer (0.3 M) for 2 hr at 37°C. Released glucose was quantified by the spectrophotometric measurement of NADH production (λ = 340 nm) in the presence of hexokinase (H4502 Sigma) and glucose‐6‐phosphate dehydrogenase (G6378 Sigma), according to the method of Bergmeyer (Bergmeyer & Grassl, 1983; Passonneau & Lauderdale, 1974) and as previously described in (Banzet et al., 2009). Glycosyl units were quantified by comparison to a standard curve of known glycogen concentration.

#### 2.5.3 Analysis of protein content by western blotting

Muscle samples from the soleus were homogenized in a 1/100 dilution using an extraction buffer containing 0.05 M Tris‐HCl (T 5941), 0.1 M NaCl, 2 mM EDTA (E5134), 2 mM EGTA (E4378), 1 mM DTT (D0632), 0.05 M NaF (S7920), 0.12 µM okadaic acid (O9381, 3 mM benzamidine (B5606), 0.05 M glycerophosphate (G5422), and 1 mM PMSF (P7626, Sigma‐Aldrich), with the addition of an anti‐protease cocktail at 1/200 and an anti‐phosphatase cocktail at 1/200 (539134‐1S and 524625‐1S, respectively, Merck Chemicals), and shaken in a Precellys^®^ as described above for enzymatic activity.

**FIGURE 3:**
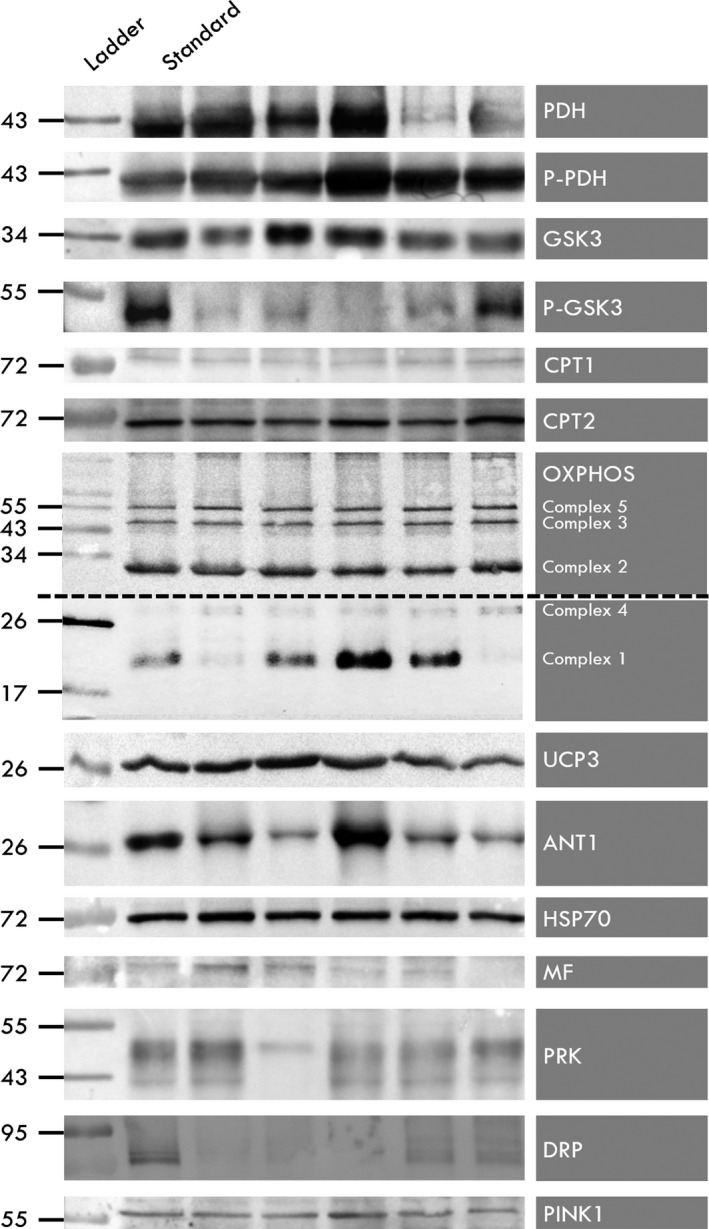
Typical western blotting analysis illustrated for each measured protein. The muscle cells lysate was obtained from each animal of the four groups (Control, heat acclimated, trained, and heat + trained) in the presence of protease and phosphatase inhibitors. The proteins were resolved by SDS‐PAGE (7.5%–12%) and electroblotted onto a nitrocellulose membrane. Membranes were probed with primary antibody (Table 1) and then peroxidase‐tagged with secondary antibody (anti‐mouse or anti‐rabbit, 1/10,000). Membranes were soaked in ECL solution and scanned using a ChemiDoc XRS+ (Bio‐Rad). A molecular‐weight ladder (Precision Plus All Blue Prestained Protein Standards from Bio‐Rad Laboratories, no. 1610373) and an internal control (mix of all control group animals) were also loaded onto each gel. The bands corresponding to the molecular weight of the proteins of interest were quantified using Quantity One (version Windows, Bio‐Rad) and normalized to the internal control (software ImageLab). Dash line brands the delimitation between the two pictures of the same gel performed at two different development times (1 and 60 s) to get a good definition of each lane and subsequent quantification for the five OXPHOS complexes. PDH; pyruvate dehydrogenase. P‐PDH: phosphorylated PDH (E1 subunit). GSK, glycogen synthase kinase; P‐GSK, phosphorylated GSK; CPT, carnitine palmitoyltransferase/UCP‐3, uncoupling protein 3; ANT‐1, adenine nucleotide translocase 1; HSP‐70, heat shock protein 70; MF, mitofusin, PRK, parkin; DRP, dynamin‐related protein; PINK, PTEN‐induced putative kinase

Samples were cooled on ice for 12 hr and then centrifugated at 12,000 ***g*** for 20 min at 4°C. The protein concentration was quantified using the bicinchoninic acid method (kit BCA Protein Assay 23252 Thermo Pierce) according to the manufacturer's protocol. Equal amounts of protein (25 µg) were loaded onto 7.5%–12% polyacrylamide SDS‐PAGE gels, separated, and transferred to nitrocellulose membranes using a transfer apparatus according to the manufacturer's protocols (Bio‐Rad). Membranes were blocked by incubation in 5% nonfat milk in TBST (25 mM Tris, pH 7.5, 137 mM NaCl, 2.68 mM KCl, 0.5% Tween 20) for 2 hr. Membranes were incubated at 4°C overnight with primary antibodies listed in Table 1. For phosphorylated protein, 50 mM NaF (S7920 Sigma) was added at each step. Membranes were washed three times for 10 min in TBST and incubated with a 1:10,000 dilution of secondary antibodies (horseradish peroxidase‐conjugated anti‐mouse or anti‐rabbit antibodies) for 1 hr. Blots were washed with TBST three times for 10 min in TBST and developed using the ECL system (Bio‐Rad) according to the manufacturer's protocols. Blots were scanned using a ChemiDoc XRS+ (Bio‐Rad). A molecular‐weight ladder (Precision Plus All Blue Prestained Protein Standards from Bio‐Rad Laboratories, no.1610373) and an internal control (mix of all control group specimens) were also loaded onto each gel. The bands corresponding to the molecular weight of the proteins of interest were quantified using Quantity One (version Windows, Bio‐Rad) and normalized to the internal control (Image Lab software) (Figure 3).

**TABLE 1:** Reference and conditions of the utilization of primary antibodies

	Antibodies reference	% acrylamide in gel	Dilution antibody	HRP‐conjugated secondary antibody	ECL substrate
Anti‐Pyruvate Dehydrogenase E1‐alpha subunit (phospho S293) antibody [EPR12200]	ab177461 Abcam	10%	1/1000	Anti‐rabbit antibodies ab6721 Abcam	Clarity WB ECL Bio‐Rad
Anti‐Pyruvate Dehydrogenase E1‐alpha subunit antibody [EPR11098]	ab168379 Abcam	10%	1/1000	Anti‐rabbit antibodies ab6721 Abcam	Clarity WB ECL Bio‐Rad
Total OXPHOS Human WB Antibody Cocktail	ab110411 Abcam	15%	1/400	anti‐mouse antibodies ab205719 Abcam	Clarity WB ECL Bio‐Rad
Anti‐Adenine Nucleotide Translocase 1/ANT 1 antibody	ab110322 Abcam	10%	1/1000	Anti‐mouse antibodies ab205719 Abcam	Clarity WB ECL Bio‐Rad
Anti‐CPT1A antibody	ab128568 Abcam	10%	1/500	Anti‐mouse antibodies ab205719 Abcam	Clarity Max WB ECL Bio‐Rad
Anti‐CPT2 Antibody	ABS85 Abcam	10%	1/1000	Anti‐rabbit antibodies ab6721 Abcam	Clarity WB ECL Bio‐Rad
Anti‐Uncoupling Protein 3 Antibody	AB3046 Abcam	10%	1/1000	Anti‐rabbit antibodies ab6721 Abcam	Clarity WB ECL Bio‐Rad
HSP70 Antibody	4872S Ozyme	10%	1/1000	Anti‐rabbit antibodies ab6721 Abcam	Clarity WB ECL Bio‐Rad
Phospho‐GSK‐3alpha/beta (Ser21/9) Antibody	9331S Ozyme	10%	1/500	Anti‐rabbit antibodies ab6721 Abcam	Clarity WB ECL Bio‐Rad
GSK‐3beta (27C10) Rabbit mAb	9315S Ozyme	10%	1/500	Anti‐rabbit antibodies ab6721 Abcam	Clarity WB ECL Bio‐Rad
Parkin (PrK8) Mouse mAb	4211S Cell signaling	10%	1/500	Anti‐mouse antibodies ab205719 Abcam	Clarity WB ECL Bio‐Rad
DRP1 (D8H5) Rabbit mAb	5391S Cell signaling	10%	1/500	ANTI‐rabbit antibodies ab6721 Abcam	Clarity WB ECL Bio‐Rad
Mitofusin‐2 (D2D10) Rabbit mAb	9482S Cell signaling	10%	1/500	Anti‐rabbit antibodies ab6721 Abcam	Clarity WB ECL Bio‐Rad
Anti‐PINK1 antibody	Ab23707 Abcam	10%	1/500	Anti‐rabbit antibodies ab6721 Abcam	Clarity WB ECL Bio‐Rad

### 2.6 Measurement of in situ mitochondrial respiration

Measurements were performed in tightly temperature‐controlled chambers at 35°C. Mitochondrial respiration was studied in situ using a Clarke‐electrode (Hansatech Oxygraph Instruments), as previously described Kuznetsov et al. (2008). Freshly taken muscles were stored and the fibers separated under a binocular microscope in solution S (see below for composition) at 4°C. Fibers were then permeabilized in solution S with saponin (50 µg.ml^−1^) for 30 min and stored 15 min in solution S, always at 4°C. Before respiration measurements, some fibers were rinsed three times for 5 min in solution R (see below for composition), containing no energy source for respiration, thus washing out any remaining energetic substrates, such as adenosine diphosphate (ADP) or creatine phosphate (PCr). A small quantity of fibers was placed in the well of an oxygraph containing 1.5 ml of solution R with continuous stirring. The decrease in O_2_ concentration in the well reflected fiber respiration and the slope represented the rate of oxygen consumption. For pyruvate oxidation measurements, 2 mM pyruvate and 4 mM malate were added to the respiration media and for PCoA, 0.4 mM palmitoyl‐CoA, 0.5 mM malate, and 2 mM carnitine were added. Thus, we measured the non‐phosphorylating respiration rate (Pyr‐${\dot{V}}_{0}$ and PCoA‐${\dot{V}}_{0}$) in the absence of ADP. Then, 2 mM ADP was added to obtain a non‐limiting concentration for mitochondrial respiration (Martins et al., 2018; Ojuka et al., 2016; Pesta & Gnaiger, 2012; Ponsot et al., 2006; Zoll et al., 2002). The negative slope of the respiration curve during this phase corresponded to the maximal respiration rate for Pyr and PCoA (Pyr‐${\dot{V}}_{\text{max}}$ and PCoA‐${\dot{V}}_{\text{max}}$). The slope was measured once the reaction stabilized, first after substrate addition and then after ADP addition.

After completing the measurements, the fibers were dried and weighed to obtain the respiration results in µmol O_2_ min^−1^ g^−1^ dry weight. Cytochrome C (cyt C) was systematically added after the last addition of substrate and before that of the inhibitor to verify the preservation of the mitochondrial outer membrane. The results were not considered if cytochrome C induced an increase in the rate of respiration >10% of the previous rate. Measurements were performed in duplicate for each sample. We calculated the respiratory control ratio ($\text{RCR}={\dot{V}}_{\text{max}}/{\dot{V}}_{0}$) for both substrates (PCoA‐RCR and Pyr‐RCR).

Solutions S and R, pH 7.1, both contained 2.77 mM CaK_2_ EGTA, 7.23 mM K_2_ EGTA, 6.56 mM MgCl_2_ (1 mM free Mg^2+^), 20 mM taurine, 0.5 mM DTT, and 50 mM K‐methane sulfonate (160 mM ionic strength). Solution S also contained 3 mM phosphate and 2 mM ADP (Martins et al., 2018; Ponsot et al., 2006; Zoll et al., 2002). Bovine serum albumin (BSA) was added to solution R just before the respiration measurements (Oliveira et al., 2015; Ou & Leiter, 2004). For the Pyr media, the BSA concentration was 2 mg ml^−1^. For the PCoA media, the BSA concentration in R was 6 mg ml^−1^ (ratio 4.4) to obtain a FA/albumin ratio <5 and to prevent any detersive effects of the FAs. This concentration was determined in a pilot study in which various BSA concentrations (2, 4, and 6 mg ml^−1^) were tested.

### 2.7 Statistical analysis

Data are presented as the means ± SEM of values per animal, from either the mean of duplicates or one sole value after data validation. Two‐way ANOVA was performed to assess the effect of heat acclimation and training. When appropriate, comparisons were performed between groups using the Newman‐Keuls test. Differences were considered significant when *p < .05, **p < .01, or ***p < .001 for heat acclimation and ^$^ *p* < .05, ^$$^ *p* < .01, or ^$$$^ *p* < .001 for training.

## 3 RESULTS

### 3.1 Anthropometric and resting metabolic data

After 6 weeks, the body weight and adipose tissue mass of the T groups were less than those of the sedentary groups, independently of heat acclimation (−43% of the retroperitoneal fat mass, *p* < .001), whereas there was no difference in the mass of the soleus muscle (Table 2).

**TABLE 2:** Body and tissue weight in male rats after 6‐week conditioning

Weight (g)	C (8)	H (8)	T (7)	H+T (8)	
Initial body	277.88 ± 11.20	286.25 ± 12.27	269.14 ± 8.89	270.50 ± 11.79	
Final body	395.25 ± 6.86	386.75 ± 10.52	361.71 ± 6.90	364.25 ± 6.81	Training effect (*p* < .001)
Soleus	0.16 ± 0.01	0.15 ± 0.01	0.14 ± 0.02	0.13 ± 0.01	
Adipose tissue	8.19 ± 0.68	7.86 ± 0.85	4.22 ± 0.34	4.97 ± 0.52	Training effect (*p* < .001)

Note

Data are expressed as the means ± SEM per group (*n*). Analyses of variance (ANOVA) was performed to compare the effect of two factors (training, heat acclimation) for each weight. A training effect was observed without interaction with heat acclimation.

Abbreviations: C, control; H, heat acclimated; T, endurance trained; H+T, heat acclimated and trained.

Glycerol and free fatty‐acid concentrations in the plasma were similar but resting glycemia was significantly lower for the T groups (−22%, *p* < .001) than for the sedentary animals (Table 3). Nevertheless, the resting metabolic rate (O2 consumption) and fuel utilization (assessed through RQ) were no different (Table 4).

**TABLE 3:** Resting blood parameters in male rats after 6‐week conditioning

	C	H	T	H+T	
Glucose (g/L)	2.12 ± 0.07 (7)	2.14 ± 0.16 (8)	1.58 ± 0.07 (7)	1.77 ± 0.14 (8)	Training effect (*p* = .001)
Glycerol (µmol/L)	65.92 ± 4.48 (7)	104.97 ± 20.35 (8)	60.03 ± 5.49 (7)	62.45 ± 10.48 (8)	
NEFA (mmol/L)	0.14 ± 0.02 (7)	0.21 ± 0.06 (7)	0.12 ± 0.02 (6)	0.09 ± 0.01 (6)	

Note

Data are expressed as the means ± SEM per group (*n*). ANOVA was performed to compare the effect of two factors (training, heat acclimation) for each concentration. A training effect was observed without interaction with heat acclimation.

Abbreviations: C, control; H, heat acclimated; T, endurance trained; H+T, heat acclimated and trained.

**TABLE 4:** Maximal aerobic speed and resting indirect calorimetry measurements in male rats after 6‐week conditioning

	C (8)	H (8)	T (7)	H+T (8)	
MAS (m/min)	25.26 ± 1.59	28.30 ± 1.08	49.10 ± 1.35	47.16 ± 2.23	Training effect (*p* < .001)
RQ	0.85 ± 0.01	0.87 ± 0.01	0.86 ± 0.01	0.85 ± 0.01	
VO2 (ml/min/kg^0.75)	13.88 ± 0.46	14.24 ± 0.26	14.81 ± 0.22	14.53 ± 0.33	
VCO2 (ml/min/kg^0.75)	11.84 ± 0.47	12.39 ± 0.28	12.75 ± 0.15	12.35 ± 0.31	

Note

Data are expressed as the means ± SEM per group (*n*). ANOVA was performed to compare the effect of two factors (training, heat acclimation) for each variable. A training effect was observed without interaction with heat acclimation.

Abbreviations: MAS, maximal aerobic speed; RQ, respiratory quotient; VO_2_, dioxygen consumption; VCO_2_, carbon dioxide production; C, control; H, heat acclimated; T, endurance trained; H+T, heat acclimated and trained.

### 3.2 Performance and heat acclimation data

As expected, the maximal aerobic speed MAS was increased by endurance training (+80%, *p* < .001) but without any interaction with heat acclimation (Table 4).

More surprisingly, HSP70 protein content in the soleus muscle was not increased either by training or by heat exposure (Table 6).

### 3.3 Oxidative capacity and mitochondrial content and dynamics of muscle

Not surprisingly, citrate synthase activity, considered to be a good marker of mitochondrial mass in the soleus muscle, increased by 54% (*p* < .001) following training, with no effect of/or interaction with heat acclimation (Table 5).

**TABLE 5:** Maximal activities of citrate synthase (CS), 3‐hydroxyl acyl coenzyme A dehydrogenase (3‐HAD), hexokinase (HK) and lactico‐deshydrogenase (LDH) of soleus muscle after 6‐week conditioning of male rats

	C	H	T	H+T	
Enzyme activity UI/g fresh tissue					
CS	22.20 ± 1.28 (7)	26.82 ± 1.67 (8)	37.64 ± 1.85 (6)	37.61 ± 2.68 (8)	Training effect *p* < .001
HAD	24.40 ± 0.58 (8)	24.50 ± 1.23 (7)	27.10 ± 1.38 (7)	28.32 ± 1.38 (8)	Training effect *p* < .05
HK	0.89 ± 0.19 (7)	0.84 ± 0.14 (8)	1.03 ± 0.07 (6)	0.87 ± 0.17 (7)	
LDH	135.50 ± 16.97 (8)	144.16 ± 6.64 (8)	111.44 ± 11.71 (7)	129.30 ± 5.80 (8)	Training effect *p* < .05

Note

Data are expressed as the means ± SEM. UI per g of fresh tissue. Control (C), heat acclimated (H), trained (T) and heat acclimated and trained (H+T). Two‐factor ANOVA was performed to compare the effect of heat and training on protein content. There was no interaction between heat‐acclimation and training effects.

Protein content of the various respiratory chain complexes (electron transport chain: complexes I, III, and IV and ATP synthase: complex V) did not significantly increase with training. There was an interaction between heat acclimation and training for cytochrome C oxidase content (complex IV), which was higher in the T+H group than the T only group (fivefold higher, *p* < .05) (Table 6).

**TABLE 6:** Expression of proteins involved in carbohydrates and fatty acids mitochondrial metabolism, proteins of the mitochondrial respiratory chain (I, II, III, IV, and V complexes), putative cytoprotective proteins and proteins involved in mitochondrial remodeling in soleus after 6‐week conditioning of male rats

	C	H	T	H+T	
Lipids and CHO oxidation steps					
PDH	0.82 ± 0.15 (8)	0.64 ± 0.14 (8)	1.08 ± 0.24 (7)	1.01 ± 0.16 (8)	Training effect (*p* = .057)
P‐PDH	1.01 ± 0.15 (8)	1.07 ± 0.15 (8)	1.25 ± 0.19 (7)	1.22 ± 0.18 (8)	
GSK	1.12 ± 0.18 (8)	1.20 ± 0.20 (8)	1.20 ± 0.15 (7)	0.89 ± 0.22 (8)	
P‐GSK	0.25 ± 0.10 (6)	0.22 ± 0.04 (7)	0.36 ± 0.11 (7)	0.22 ± 0.04 (8)	
CPT‐1	0.77 ± 0.16 (8)	0.67 ± 0.19 (8)	0.90 ± 0.07 (7)	0.96 ± 0.26 (8)	
CPT‐2	0.89 ± 0.08 (8)	0.75 ± 0.05 (7)	0.47 ± 0.10 (7)	0.74 ± 0.06 (7)	Heat acclimation effect (*p* < .05)
Mitochondrial respiratory chain					
CI	0.91 ± 0.21 (8)	1.10 ± 0.16 (7)	1.27 ± 0.18 (6)	1.20 ± 0.08 (8)	
CII	0.84 ± 0.21 (8)	0.99 ± 0.15(7)	1.30 ± 0.20(6)	1.13 ± 0.11 (8)	
CIII	1.16 ± 0.09 (7)	1.12 ± 0.12 (7)	1.27 ± 0.06 (7)	1.10 ± 0.05 (8)	
CIV	0.28 ± 0.04 (8)	0.11 ± 0.03 (8)	0.16 ± 0.04 (7)	0.85 ± 0.32 (8)	Heat and training effect (*p* < .05)
CV	1.70 ± 0.53 (6)	2.19 ± 0.44 (8)	2.14 ± 0.41 (7)	1.44 ± 0.25 (8)	
Putative cytoprotection					
UCP‐3	1.01 ± 0.13 (8)	0.96 ± 0.16 (8)	1.07 ± 0.12 (7)	1.04 ± 0.18 (7)	
ANT‐1	0.50 ± 0.07 (8)	0.65 ± 0.18 (8)	0.66 ± 0.14 (7)	0.56 ± 0.06 (8)	
HSP‐70	1.00 ± 0.10 (8)	0.93 ± 0.03 (6)	1.25 ± 0.15 (7)	1.14 ± 0.17 (7)	
Mitochondrial remodeling					
MF	0.63 ± 0.15 (7)	0.38 ± 0.04 (6)	0.77 ± 0.18 (7)	0.86 ± 0.26 (8)	
PRK	1.55 ± 0.20 (8)	1.33 ± 0.25 (8)	0.85 ± 0.10 (7)	0.84 ± 0.22 (8)	Training effect (*p* < .001)
DRP	0.73 ± 0.14 (8)	0.60 ± 0.14 (8)	0.43 ± 0.12 (7)	0.34 ± 0.11 (8)	Training effect (*p* < .05)
PINK	0.66 ± 0.12 (7)	0.68 ± 0.16 (7)	0.47 ± 0.10 (7)	0.47 ± 0.12 (8)	

Note

Data are expressed as the means ± SEM in fold standard. Control (C) heat acclimated (H) trained (T), and heat acclimated and trained (H+T). Two‐factor ANOVA was performed to compare the effect of heat and training on protein content. There was no interaction between heat‐acclimation and training effects.

Abbreviations: PDH, pyruvate dehydrogenase; P‐PDH, phosphorylated PDH (E1 subunit); GSK, glycogen synthase kinase; P‐GSK, phosphorylated GSK; CPT, carnitine palmitoyltransferase; UCP‐3, uncoupling protein 3; ANT‐1, adenine nucleotide translocase 1; HSP‐70, heat shock protein 70; MF, mitofusin; PRK, parkin; DRP, dynamin‐related protein; PINK, PTEN‐induced putative kinase.

Cellular Parkin (*p* < .01) and DRP‐1 (*p* < .05) proteins, markers of mitophagy and mitochondrial fission, respectively, were lower in the trained than untrained groups, without any interaction with heat acclimation. No change was found for mitofusin, a marker of mitochondrial fusion and PINK, a marker linked to the activation of autophagy by damaged mitochondria (Table 6).

### 3.4 Glycolytic flux and carbohydrate oxidation

**FIGURE 4:**
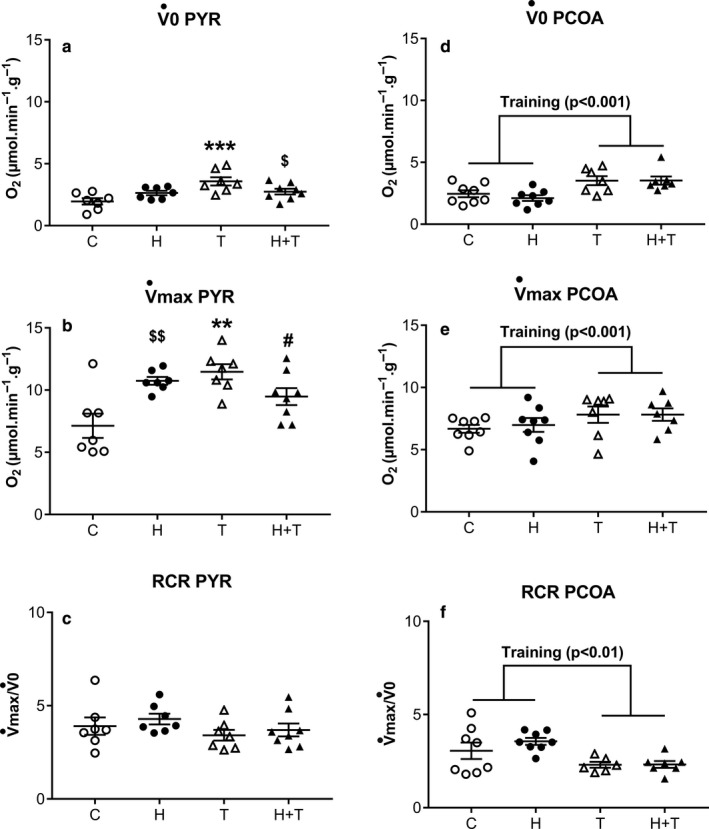
The effect of heat acclimation and endurance training on in situ mitochondrial respiration in permeabilized soleus muscle fibers of male rats. Respiration rates were measured in the absence (${\dot{V}}_{0}$) or presence of ADP (${\dot{V}}_{\text{max}}$), for palmitoyl‐coenzyme A + carnitine and pyruvate substrates. Non‐phosphorylating (${\dot{V}}_{0}$) and phosphorylating (${\dot{V}}_{\text{max}}$) rates and the respiratory control ratio (RCR), corresponding to the ${\dot{V}}_{\text{max}}/{\dot{V}}_{0}$ ratio in the control group (open circles), heat‐acclimated group (closed circles), trained group (open triangles), and heat‐acclimated and trained group (closed triangles) for pyruvate (A,B,C) and PCoA (D,E,F) are presented. Each point shows the value of one animal. The lines represent the means for all animals ± SEM. An analysis by two‐factor ANOVA was performed to compare the effect of heat and training on the respiration parameters of both substrates. *Post*‐*hoc* analysis was performed using the Newman‐Keuls test. **p < .01, ***p < .001: difference from the heat exposure‐matched group. ^$^ *p* < .05, ^$$^ *p* < .01: difference from the training‐matched group. # difference from the control group

As expected, the ${\dot{V}}_{0}$ and ${\dot{V}}_{\text{max}}$ with pyruvate increased in the soleus muscle following training (*p* < .005 and *p* < .05, respectively). There was an interaction of training with heat acclimation (interaction *p* < .01 for Pyr‐${\dot{V}}_{0}$ and *p* < .001 for Pyr‐${\dot{V}}_{\text{max}}$) such that we only observed the effect of training on pyruvate oxidation in the group that was not exposed to heat. The increase in Pyr‐${\dot{V}}_{0}$ following endurance training was thus limited when combined with heat acclimation, +80% in the non‐heat‐acclimated groups vs +4% in the heat‐acclimated groups, resulting in a Pyr‐${\dot{V}}_{0}$ in the H+T lower than in T groups (−23%; *p* < .05). Pyr‐${\dot{V}}_{\text{max}}$ in the H and H+T groups was significantly higher than for the C group (+50%, *p* < .005 and +30%, *p* < .05, respectively), but with no synergy between heat acclimation and endurance training (Figure 4).

**FIGURE 5:**
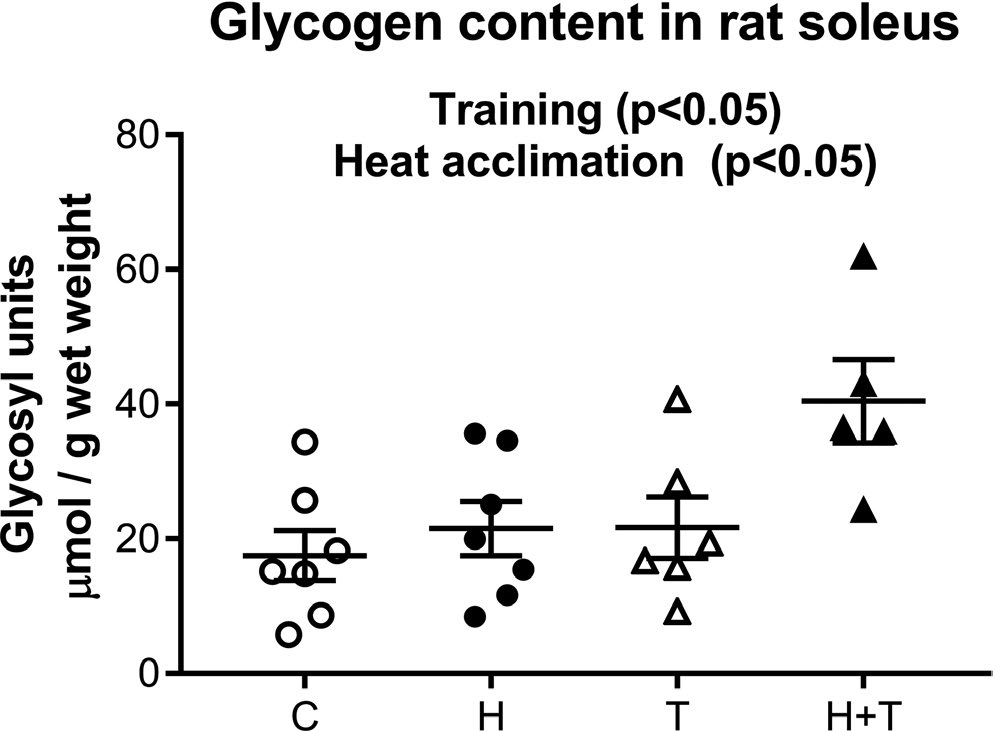
The effect of heat acclimation and endurance training on glycogen content in soleus muscle of male rats. C: control group (open circles), H: heat‐acclimated group (closed circles), T: trained group (open triangles), H+T: heat‐acclimated and trained group (closed triangles). Data are expressed in µmol of glycosyl units g^−1^ wet weight. Each point shows the value of one animal. The lines represent the means for all animals ± SEM. An analysis by two‐factor ANOVA was performed to compare the effect of heat and training on the respiration parameters of both substrates

As expected, there was a global effect of training on soleus muscle glycogen content (*p* < .05) but there was also an independent effect of heat acclimation (*p* = .01), with no interaction (Figure 5). This was evident for the glycogen content in the H+T group, which was twice that of the other groups. Nevertheless, there were no alterations in glycogen synthase kinase 3 (GSK3) protein levels or the ratio between the phosphorylated form (which inactivates glycogen synthase) and total protein content (Table 6). There was also no difference in HK activity between the groups. Total LDH activity was slightly decreased (15%) by training, independently of heat exposure (*p* < .05) (Table 5).

Although pyruvate dehydrogenase (PDH) content tended to increase following training (*p* = .057), we did not detect any alterations in the level of the serine‐phosphorylated form of this protein and the functional ratio of total PDH to the phosphorylated form was not altered by either training or heat acclimation (Table 6).

### 3.5 Fatty‐acid flux and oxidation

Mitochondrial oxidation of PCoA increased with training (+58%, *p* < .001 for PCoA‐${\dot{V}}_{0}$; +18%, *p* = .01 for PCoA‐${\dot{V}}_{\text{max}}$), with no effect of heat acclimation *per se* and no interaction. (Figure 4).

Similarly, HAD activity increased with training (+13%, *p* = .01) and the ratio of CS/HAD activity was unchanged (Table 5).

The protein content of CPT‐1 and complex II of the respiratory chain was unaltered by conditioning, whereas the CPT‐2 content decreased following heat acclimation (−17%, *p* < .05) (Table 6).

### 3.6 Mitochondrial quality and coupling

We found a lower Pyr‐${\dot{V}}_{\text{max}}$/CS ratio with training (*p* < .05), with no effect of heat acclimation *per se*, but a trend toward an interaction with training (*p* = .08). Pyr‐${\dot{V}}_{\text{max}}$/CS ratio was constant in the non acclimated‐group after endurance training (0.34 ± 0.6 and 0.32 ± 0.02, respectively) but trended to decrease in H+T group compared to H group (0.26 ± 0.20 vs 0.41 ± 0.4, respectively) (Figure 4, Table 6).

The Pyr‐RCR was unchanged, regardless of the condition, whereas the PCoA‐RCR decreased by 30% with training (*p* < .005) (Figure 4). Neither UCP3 nor ANT‐1 protein expression were altered by training or heat exposure and there was no difference between groups for the ratio between complex I and ATP synthase protein content (Table 6).

## 4 DISCUSSION

In this study, we assessed the effect of endurance training combined with heat acclimation on mitochondrial adaptations for the first time, considering the quantitative and qualitative alterations of carbohydrates and fatty acids oxidation.

First, we found the classical effect of endurance training on anthropometry, performance, and mitochondrial adaptations of skeletal muscle. Thus, fat mass and resting glycemia decreased. MAS increased, as well as mitochondrial content and maximal oxidation for pyruvate and PCoA.

Heat acclimation only altered endurance training effect on mitochondrial pyruvate oxidation and carbohydrate pathway. Thus, the effect of training on Pyr‐${\dot{V}}_{0}$ and Pyr‐${\dot{V}}_{\text{max}}$ was not found for the heat acclimatized group heat acclimatized as if heat acclimation had limited endurance training response on carbohydrate oxidation. However, although no heat‐acclimation effect was found *per se*, the Pyr‐${\dot{V}}_{\text{max}}$ in the H group was higher than in the C group and a bit lower but not significantly different from that of the T group.

Moreover, muscle glycogen content was increased by both training and heat exposure, with storage doubled in the group that was trained and heat acclimatized. We also observed an interaction between heat acclimation and training on the protein content of complex IV of the respiratory chain. Nevertheless, this isolated increase in protein expression was not associated with an improvement of mitochondrial respiration or efficiency.

Finally, such a metabolic phenotype of skeletal muscle after heat acclimation combined with endurance training does not appear to improve substrate oxidation more than training *per se* but may facilitate glycolytic flux.

### 4.1 Limits

#### 4.1.1 Heat acclimation phenotype

We ensured in a pilot study before this experiment that our heating protocol could induce hyperthermia by measuring core temperature with a telemetric pill (Anipill, BodyCAP). Device was implanted in the abdominal cavity of seven rats to assess the effect of the heating protocol. We observed an elevation of the core temperature above 39°C for 40 min and above 39.5°C for 23 min for all animals except one. Nevertheless, it would have been also interesting to measure core temperature during the training session. Indeed, even we have no measurement in our situation of exercise, it is well established that temperature rises considerably in rats while running on a treadmill at normal room temperature (Rodrigues et al., 2003; Taylor et al., 1999). Thus, we do not deny that training‐induced phenotype provides heat acclimation. Nevertheless, if endurance training‐induced partial heat acclimation, the phenotype is not complete and can be improved by additive heat exposure even in well‐trained athletes (Lorenzo et al., 2010). Moreover, independently of core temperature elevation and systemic heat acclimation, the elevation of muscle temperature during exercise may also be one stimulus responsible for metabolic adaptations. Yet, it was one of our hypotheses that heat could be per se a mitochondrial biogenesis driver. That is why we wanted to study the metabolic effect of passive heating exposure, independently from exercise with a group passively exposed for a duration close to exercise time. As it is hard to separate the different stimuli possibly responsible for metabolic adaptations induced by endurance training and our aim was not to assess which part of endurance training adaptation was related to heat acclimation, we studied the potential synergistic effect of heat exposure and endurance training. Thus, we included one group exposed to heat and exercise, where heat exposure was delayed from the exercise session to increase the duration of heat exposure. Increasing heat load during exercise may be tricky in rodents because of their vulnerability to heatstroke.

#### 4.1.2 Hydration status

Furthermore, even if animals were ensured free access to water and food, we cannot rule out that dehydration may have occurred and interfered in conditioning, in the combined exposition group. Only weighting was available at regular intervals and showed no intragroup disparity. Moreover, animals were sacrificed at distance from the last exercise session and heat exposure (at least 48 hr), limiting the effect of the last session on dehydration with enough time to rehydrate.

#### 4.1.3 HSP content

Surprisingly, we did not find a significant increase in HSP 70 protein content in the soleus muscle neither after heat acclimation nor after endurance training. The longer period of heat acclimation used in our study relative to that of other protocols may offer an explanation (Tamura et al., 2014). Moreover, while slow‐twitch muscle has a higher basal level of HSP, this pool is less inducible by heat, and the effect does not last as long as that in the fast‐twitch muscle (Brownstein et al., 2017; Oishi et al., 2002; Tamura et al., 2017). Other authors have not found an increase in HSP70 mRNA levels in the soleus muscle (Kodesh & Horowitz, 2010) after heat acclimation.

### 4.2 No additive effect of heat acclimation on quantitative mitochondrial adaptations induced by endurance training

As expected, endurance training induced an increase in mitochondrial oxidative capacity, as well as pyruvate and PCoA oxidation. Consistent with these findings, CS activity, which is used to assess mitochondrial mass (Larsen et al., 2012), increased in the trained groups.

Combining heat exposure after endurance training session did not improve exercise‐induced mitochondrial biogenesis. Tamura et al. (2014) have already studied the combination of endurance training and heat acclimation on respiratory complex protein content and CS activity but not mitochondrial function. Our results did not confirm such a synergistic effect of heat acclimation on mitochondrial biogenesis, possibly due to potential differences between animal species (rats here vs mice for the study of Tamura et al.) and the endurance training and heat acclimation protocols. Our results are consistent with those found at the cellular level, for which PGC‐1 α nuclear protein content, the main effective factor of mitochondrial biogenesis, was similar after exercise at room temperature or under hot conditions (Heesch et al., 2016).

Thus, it appears that heat acclimation does not enhance training‐induced adaptions of mitochondrial content.

### 4.3 The mitochondrial efficiency, quality, and dynamics observed after endurance training are not altered by heat acclimation

We observed a significant 30% decrease in the respiratory control ratio (RCR) for PCoA with training, consistent with an alteration of oxidative phosphorylation coupling. Sparks et al. (2016) showed that training‐induced increases in insulin sensitivity were associated with increased FA‐induced uncoupling. Nevertheless, such putative training‐induced uncoupling for fatty acids was not increased by heat acclimation, as we expected, because of the previously found acute effect of heat on fatty‐acid uncoupling (Tardo‐Dino et al., 2019). In our study, neither alterations of UCP‐3 nor ANT‐1 protein expression can explain such a decrease in the RCR. Our results instead suggest a functional alteration, which is consistent with the training‐induced‐uncoupling which is specific to fatty acids but not occurs with CHO.

Concerning qualitative adaptations, Kodesh et al. suggested that heat acclimation may improve the efficiency of skeletal‐muscle and heart contractility to limit heat production during contraction (Kodesh & Horowitz, 2010; Kodesh et al., 2011). Our data on the RCR showed that mitochondrial oxidative phosphorylation efficiency was not improved with heat acclimation.

Heat exposure has been suggested to impair the autophagosome complex (Brownstein et al., 2017). Although the main purpose of our study was not to study mitochondrial dynamics, we observed a decrease of the levels of DRP1, involved in mitochondrial fission (Drake et al., 2016; Tanaka et al., 2020), and of PRK, a key enzyme of the mitophagy pathway involved in senescent mitochondrial turn‐over, with training but independent of heat acclimation. The decrease of PRK suggests an alteration of the maintenance of mitochondrial quality in response to endurance training. These results are not in accordance with the acute effect of exercise described in other studies (Faure et al., 2016; Zoladz & Grassi, 2015) but our results must be interpreted with caution because we only measured total protein content in whole muscle.

Whatever, our results did not argue for specific alterations of mitochondrial quality with heat acclimation, while mitochondrial substrate oxidation appears to have been differently affected by training, depending on the state of heat acclimation.

### 4.4 The effect of heat acclimation combined with endurance training on mitochondrial oxidation depends on the nature of the substrate that is oxidized

PCoA‐${\dot{V}}_{0}$ and PCoA‐${\dot{V}}_{\text{max}}$ were similar in the H+T group and in the T group (−7% and −5%, respectively, ns). Thus, PCoA oxidation rates increased in both trained groups, heat acclimated or not. HAD activity was increased by endurance training with a similar effect size as that of the PCoA‐${\dot{V}}_{\text{max}}$ (+13% and +18%, respectively). Although CPT‐1 protein content did not increase and that of CPT‐2 decreased by 17% following heat acclimation, we could interpret this result as CPT‐1 and CPT‐2 content are not limiting factors in the soleus muscle for this magnitude of increase in PCoA oxidation.

Conversely, the Pyr‐${\dot{V}}_{0}$ and Pyr‐${\dot{V}}_{\text{max}}$ of the H+T group were 23% (*p* < .05) and 17% (ns) lower, respectively, than those of the T group. At the same time, we measured 40% greater CS activity in the H+T group than in the H group, whereas the Pyr‐${\dot{V}}_{\text{max}}$ did not increase and even decreased by 12% (ns), resulting in a 36% decrease of the Pyr‐${\dot{V}}_{\text{max}}$/CS ratio (*p* < .01 by *T* test). Compared to the C group, the 65% increase in CS activity in the T group is consistent with the increase of the Pyr‐${\dot{V}}_{\text{max}}$ of 60%, whereas the Pyr‐${\dot{V}}_{\text{max}}$ increased by only 30% in the H+T group for the same increase of CS as in the T group. These findings argue in favor of a specific limiting factor of heat acclimation concerning pyruvate oxidation that is independent of mitochondrial mass or biogenesis. Although we did not observe any alterations of PDH protein content or that of the phosphorylated form in the T+H group, we cannot definitively rule out that pyruvate entrance into the mitochondria may be a limiting factor. This could be examined by adding dichloroacetate to the respiratory medium with pyruvate to assess the functionality of this step in future studies.

### 4.5 A putative specific heat effect on CHO metabolism in skeletal muscle

Heat exposure may induce mitochondrial biogenesis, as suggested in previous in vitro and in vivo studies (Liu & Brooks, 2012; Tamura et al., 2014). Although we did not find a global effect of heat acclimation on citrate synthase activity and pyruvate oxidation, the Pyr‐${\dot{V}}_{0}$ and Pyr‐${\dot{V}}_{\text{max}}$ in the H group were 33% (*p* = .07) and 50% (*p* < .05) higher, respectively, than in the C group. Our results only showed such a heat effect for pyruvate oxidation (significant for Pyr‐${\dot{V}}_{\text{max}}$), but not PCoA, and it was stronger than the concomitant observed increase in CS activity (+20%, ns). Overall, our results do not allow us to ascertain whether heat has a true effect on mitochondrial biogenesis and carbohydrate oxidation and such an effect in human skeletal muscle is still the subject of debate (Zak et al., 2017).

Here, glycogen storage was the highest in the H+T group, reaching a level that was twofold higher than that of the groups that only trained or were subjected to heat acclimation. Acute heat stress is well known to increase glycogen use during exercise with, especially, increased the participation of glycolysis (Febbraio et al., 1994; Febbraio et al., 1994; Fink et al., 1975; Hargreaves et al., 1996). Our results are the first to show an increase in glycogen content in skeletal muscle after chronic exposure combining endurance training and heat acclimation. This increase was not associated with a corresponding elevation of the pyruvate oxidation capacity in the H+T group, which argues for the facilitation of glycolysis. Shani et al. (Alexander‐Shani et al., 2017) described the same phenotype in heart with increased glycogen content and pyruvate dehydrogenase kinase‐1 levels (decreasing PDH activity) after heat acclimation. Kodesh et al. have already described the upregulation of genes encoding glycolysis rate‐limiting enzymes in the soleus muscle of trained and heat‐exposed rats {Kodesh, 2010 #725}but we were unable to show any heat‐induced alteration of HK or total LDH activity. We missed to measure other markers of the glycolysis pathway, such as LDH isoforms or phosphofructokinase activity. Moreover, because mitochondrial measurements were our priority, it was not possible to directly measure glycogen storage before and after the aerobic speed trial to prove our hypothesis but it would have been informative to compare the lactate concentration at the end of this trial in activity‐matched groups with a similar level of performance. To precise this putative shift toward a more glycolytic phenotype, it would have been relevant to add in our respirometry experiment the study of Km for ADP in lack and in presence of creatine to better characterize the shift in metabolic phenotype in soleus muscle.

In our study, heat acclimation was applied a few hours after the exercise session. A recent study showed that passive muscle heating during recovery can improve the glycogen resynthesis rate after depletion (Cheng et al., 2017). Glycogen synthesis is regulated by the activity of glycogen synthase kinase (GSK‐3). Although GSK‐3 can be downregulated by heat (Moon et al., 2003), we found no alteration of phosphorylated‐Ser GSK‐3 (the inactive form) protein content in the H+T group. Other explanations are possible and systemic hormonal effects of heat may increase glucose availability during the recovery period (Faure et al., 2016). The addition of such an effect on the increase in insulin sensitivity described after heat acclimation in skeletal muscle (Gupte et al., 2009) would facilitate glucose uptake (Koshinaka et al., 2013) and glycogenesis. Moreover, independently of the action of insulin, heat could *per se* activate the PI3K/Akt pathway (Liu & Brooks, 2012; Moon et al., 2003), the main anabolic pathway for protein synthesis, as well as that for glycogen resynthesis.

### 4.6 Heat‐induced hypoxia‐like adaptations of metabolism

The increase of muscle glycogen content not associated with a proportional elevation of pyruvate oxidation appears to be similar to a metabolic phenotype previously described for hypoxia (Aragonés et al., 2008; Papandreou et al., 2006) and glycolytic pathway (Semenza, 2012).

This does not mean that hypoxic stimulus is responsible for the alteration observed after heat acclimation, even some cellular argument could support the participation of hypoxia‐inducible factor (HIF) in response to cellular protection induced by heat (Liu et al., 2007; Liu & Semenza, 2007). The interaction between heat acclimation and endurance training for complex IV protein content we found here, may support this hypothesis because this respiratory complex is particularly sensitive to hypoxia and changes in its isoforms have been well described through the activation of HIF‐dependant pathways (Desplanches et al., 2014; Fukuda et al., 2007).

## 5 CONCLUSION

Our results show, for the first time, that heat acclimation does not improve mitochondrial adaptations induced by endurance training in slow‐twitch skeletal muscle, in contrast to our hypothesis. Heat acclimation combined with endurance training has no benefit on mitochondrial content or substrate oxidation or coupling. Nevertheless, the interaction found here between endurance training and heat acclimation concerning CHO metabolism and glycogen storage suggests that this association could be beneficial for the glycolytic pathway. This addresses the issue of the management of endurance training combined with heat exposure in recovery for all sportive competition requiring high glycogen storage and glycolytic flux and if it could be an alternative to repeated sprints in hypoxia strategy.

## CONFLICT OF INTEREST

No conflicts of interest, financial or otherwise, are declared by the authors.

## AUTHOR CONTRIBUTIONS

PETD and AM conceived and designed the research. PETD, AM, CT, JS, SBo, and SBa performed the experiments. PETD, AM, CT, and JS analyzed the data and interpreted the results of the experiments. PETD and AM wrote the first draft of the manuscript. PETD, NK, and AM edited and revised the manuscript. All authors approved the final manuscript.
